# Retrospective Study of Incidence and Prognostic Significance of Eosinophilia after Allogeneic Hematopoietic Stem Cell Transplantation: Influence of Corticosteroid Therapy

**DOI:** 10.4274/tjh.2015.0047

**Published:** 2016-08-19

**Authors:** Wataru Yamamoto, Eriko Ogusa, Kenji Matsumoto, Atsuo Maruta, Yoshiaki Ishigatsubo, Heiwa Kanamori

**Affiliations:** 1 Kanagawa Cancer Center, Department of Hematology, Yokohama, Japan; 2 Yokohama City University Graduate Faculty of Medicine, Department of Internal Medicine and Clinical Immunology, Yokohama, Japan

**Keywords:** Eosinophilia, Allogeneic hematopoietic stem cell transplantation, Corticosteroid therapy, Prognostic factor, graft-versus-host disease

## Abstract

**Objective:**

The clinical significance of eosinophilia after allogeneic hematopoietic stem cell transplantation is controversial. This study aimed to retrospectively study the impact of eosinophilia on the outcome of allogeneic hematopoietic stem cell transplantation by taking into account the influence of corticosteroid therapy.

**Materials and Methods:**

We retrospectively studied 204 patients with acute myeloid leukemia, acute lymphoblastic leukemia, and myelodysplastic syndrome who underwent allogeneic hematopoietic stem cell transplantation from January 2001 to December 2010.

**Results:**

The median age was 43 years (minimum-maximum: 17-65 years). Myeloablative conditioning was used in 153 patients and reduced intensity conditioning was employed in 51 patients. Donor cells were from bone marrow in 132 patients, peripheral blood in 34, and cord blood in 38. Eosinophilia was detected in 71 patients and there was no significant predictor of eosinophilia by multivariate analysis. There was no relationship between occurrence of eosinophilia and the incidence or grade of acute graft-versus-host disease when the patients were stratified according to corticosteroid treatment. Although eosinophilia was a prognostic factor for 5-year overall survival by univariate analysis, it was not a significant indicator by multivariate analysis.

**Conclusion:**

These results suggest that the clinical significance of eosinophilia in patients receiving allogeneic hematopoietic stem cell transplantation should be assessed with consideration of systemic corticosteroid administration.

## Abstract (tr)

**Amaç:**

Allojenik hematopoetik kök hücre nakli sonrası eozinofilinin önemi tartışmalıdır. Bu çalışma kortikosteroid tedavisinin etkisini hesaba katarak, eozinofilinin allojenik hematopoetik kök hücre naklinin sonuçları üzerine etkisini geriye dönük değerlendirmeyi amaçladık.

**Gereç ve Yöntemler:**

Ocak 2001’den Aralık 2010’a kadar akut myeloid lösemi, akut lenfoblastik lösemi ve myelodisplastik sendrom tanısıyla allojenik hematopoetik kök hücre nakli olan 204 hastayı geriye dönük değerlendirdik.

**Bulgular:**

Ortanca yaş 43 (aralık: 17-65 yaş) idi. Yüz elli üç hastada miyeloablatif, 51 hastada azaltılmış yoğunluklu hazırlama rejimi uygulandı. Kök hücre kaynağı 132 hastada kemik iliği, 34 hastada periferik kan ve 38 hastada kordon kanıydı. Yetmiş bir hastada eozinofili saptandı ve çoklu değişken analizinde eozinofiliyi anlamlı olarak öngörecek bir belirteç saptanmadı. Hastalar kortikosteroid tedavisine göre gruplandığında eozinofili gelişimi ile akut graft-verus-host hastalığı sıklığı ya da derecesi arasında bağlantı yoktu. Tek değişkenli analizde eozinofili 5 yıllık genel sağkalım açısından prognostik bir faktör olmasına karşın, çok değişkenli analizde anlamlı bir belirteç değildi.

**Sonuç:**

Bu sonuçlar allojenik hematopoetik kök hücre nakli olan hastalarda eozinofilinin klinik öneminin, sistemik kortikosteroid uygulamasını dikkate alarak değerlendirilmesi gerektiğinin düşündürmektedir.

## INTRODUCTION

Proliferation of eosinophils is induced by stimulation with cytokines [1] and eosinophilia occurs in various clinical settings. Eosinophilia is often found in patients receiving allogeneic hematopoietic stem cell transplantation (allo-HSCT) and a relationship between eosinophilia and the outcome and/or graft-versus-host disease (GVHD) has been reported [2,3,4,5,6,7,8,9,10]. However, the role of corticosteroid (CS) therapy should be taken into consideration with regard to evaluation of eosinophilia after allo-HSCT, because it is known that eosinophilia is influenced by such drugs [11,12]. Therefore, we retrospectively studied the impact of eosinophilia on the outcome of allo-HSCT by taking into account the influence of CS therapy.

## MATERIALS AND METHODS

Patients who underwent allo-HSCT for hematologic malignancies from January 2001 to December 2010 at the Kanagawa Cancer Center were retrospectively investigated. We defined eosinophilia as a peripheral blood eosinophil count of >500 µL on more than one occasion, while systemic steroid therapy meant CS administration at more than 0.5 mg/kg/day within 100 days after allo-HSCT. Standard-risk disease was defined as acute myeloid leukemia (AML)/acute lymphoblastic leukemia (ALL) in the first or second remission and myelodysplastic syndrome (MDS) without leukemic transformation, while high-risk disease was defined as all others. Grading of acute GVHD was done according to established criteria [13].

### Statistical Analysis

Statistical analyses were performed with R software (version 2.11.1; R Development Core Team). Differences between groups were analyzed by the Wilcoxon rank sum test or Fisher’s exact test, as was appropriate for univariate analysis and logistic regression analysis for multivariate analysis. Overall survival (OS) was calculated from the date of transplantation to the date of death from any cause or the date of last follow-up. Non-relapse mortality was defined as death without disease relapse or resistance. Time-to-event curves were drawn according to the Kaplan-Meier method and the statistical significance of differences in survival was assessed by the log-rank test. Prognostic factors included age, sex mismatch, disease risk, conditioning regimen, GVHD prophylaxis, donor type, cytomegalovirus infection, CS therapy, and eosinophilia. Either the Cox proportional hazard model or the Fine-Gray proportional hazard model was used for analysis. Death without relapse was considered to be a competing risk for relapse, relapse was a competing risk for non-relapse mortality, and relapse and death without GVHD were competing risks for GVHD.

## RESULTS

A total of 204 patients received allo-HSCT for AML, ALL, or MDS. The median follow-up period was 5.7 years and patients’ clinical characteristics are shown in Table 1. The median age was 43 years (minimum-maximum: 17-65 years) and there were 102 patients of each sex. The underlying disease was AML in 126 patients, ALL in 63, and MDS in 15. Myeloablative conditioning was used in 153 patients and reduced intensity conditioning was employed in 51 patients. Donor cells were from bone marrow in 132 patients, peripheral blood in 34, and cord blood in 38.

Eosinophilia was detected in 71 patients (34.8%). Its appearance was associated with total body irradiation (TBI), unrelated donor, and CS administration within 100 days after transplantation by univariate analysis. However, no significant predictors of eosinophilia were identified by multivariate analysis (Table 1). Among the 204 patients, 90 patients (44%) received systemic CS therapy. The reason for CS treatment was acute GVHD in 76 patients, engraftment syndrome in 4, interstitial pneumonia in 4, organizing pneumonia in 3, disease relapse in 1, diffuse alveolar hemorrhage in 1, and vasculitis in 1. The incidence of eosinophilia within 100 days after transplantation was higher in patients without CS (47/114 patients, 41.2%) than in patients with CS (24/90, 26.7%) (p=0.038). Among the 90 patients with CS, 11 were first given CS therapy after the appearance of eosinophilia. The frequency of eosinophilia was higher among patients who were not given CS therapy before eosinophilia appeared than among patients who were already receiving CS therapy (58/125 patients, 46.4% vs. 13/79, 16.5%, respectively; p<0.001).

The cumulative incidence of grade II-IV and grade III-IV acute GVHD was 45.1% and 18.6%, respectively. Table 2 shows the patients stratified according to CS treatment, GVHD grade, and occurrence of eosinophilia. There was no significant relationship between the grade of acute GVHD and occurrence of eosinophilia when patients were stratified by CS administration.

**Figure 1:**
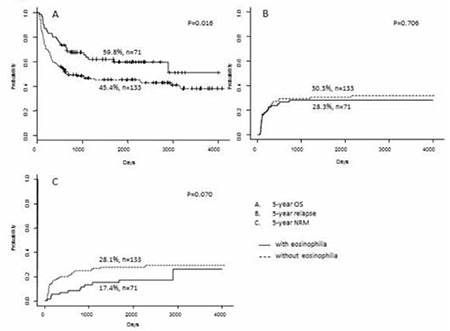
Outcome stratified according to the presence/absence of eosinophilia. A) Overall survival, B) cumulative incidence of relapse, C) non-relapse mortality.

**Figure 2:**
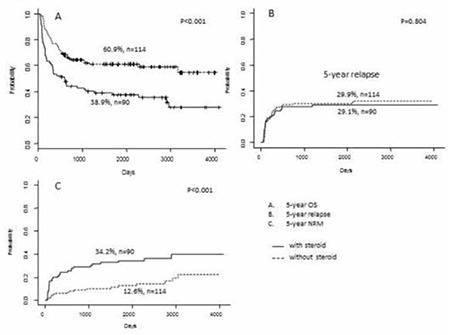
Outcomes stratified according to corticosteroid administration. A) Overall survival, B) cumulative incidence of relapse, C) non-relapse mortality.

**Figure 3:**
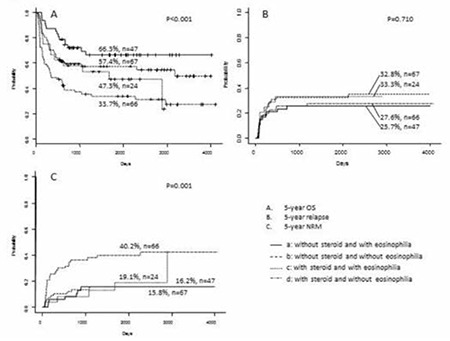
Outcomes stratified according to eosinophilia and corticosteroid therapy. A) Overall survival, B) cumulative incidence of relapse, C) non-relapse mortality.

The OS, cumulative incidence of relapse, and non-relapse mortality rate are stratified according to eosinophilia and systemic CS therapy in Figures 1 and 2, respectively. Patients with eosinophilia had a higher 5-year OS compared to those without eosinophilia (59.8%, 95% confidence interval [CI]: 48.9-73.1 vs. 45.4%, 95% CI: 37.5-55.0; p=0.016) (Figure 1A). In contrast, patients receiving CS therapy had a lower 5-year OS compared to those without CS therapy (38.9%, 95% CI: 29.9-50.7 vs. 60.9%, 95% CI: 52.3-70.9; p<0.001) (Figure 2A). However, there was no significant difference in 5-year OS between patients with or without eosinophilia among those who received CS therapy (47.3%, 95% CI: 30.3-73.9 vs. 33.7%, 95% CI: 23.9-47.7, respectively; p=0.200) (Figure 3A). Similarly, there was no difference in the 5-year OS between patients with and without eosinophilia among those not receiving CS therapy (66.3%, 95% CI: 53.6-82.1 vs. 57.4%, 95% CI: 46.5-70.8, respectively; p=0.139) (Figure 3A). Furthermore, the cumulative incidence of relapse showed no significant association with eosinophilia (Figures 1B, 2B, and 3B). However, non-relapse mortality was significantly higher in patients receiving CS therapy (Figures 2C and 3C). According to univariate analysis, eosinophilia was a good prognostic indicator for 5-year OS (hazard ratio: 0.6, 95% CI: 0.4-0.9; p=0.017), but it was not an independent prognostic indicator by multivariate analysis (hazard ratio: 0.8, 95% CI: 0.5-1.3; p=0.385). Finally, high-risk disease, unrelated donor, and CS therapy were adverse prognostic indicators for 5-year OS according to multivariate analysis (Table 3).

## DISCUSSION

In the present study, we investigated the incidence and clinical implications of eosinophilia occurring within 100 days after allo-HSCT, and we also analyzed the prognostic value of eosinophilia in relation to the influence of CS administration. The incidence of eosinophilia (34.8%) in our patient cohort was comparable with that in previous reports, although the definition of eosinophilia varies among studies. It is well known that a decrease of eosinophils is caused by CS administration [10,11]; hence, we assessed the clinical implications of eosinophilia in relation to systemic CS administration. The incidence of eosinophilia was significantly lower in patients receiving CS treatment compared with that for those without CS treatment in the present study, and the same result for patients with acute GVHD has already been described [2]. In our study, MDS, use of TBI, and transplantation from an unrelated donor were also associated with a lower incidence of eosinophilia, but the reasons for these associations are unknown.

The relationship between eosinophilia and acute GVHD after allo-HSCT remains controversial. We could not find any association between eosinophilia and acute GVHD among patients with or without CS therapy in this study. Some previous reports suggested that eosinophilia is significantly related to the onset of acute GVHD [2,9,10]. However, Aisa et al. reported that the onset of eosinophilia within 100 days after allo-HSCT is associated with a lower rate of grade II-IV acute GVHD (43% vs. 98%; p<0.001) [3]. They speculated that this association between eosinophilia and a lower incidence of severe acute GVHD may reflect the immunosuppressive effect of Th2 cytokines, which induce eosinophilia.

A relationship between the occurrence of eosinophilia after allo-HSCT and a good prognosis [2,3,4,5,6] has been reported previously. Only one study showed that there was no correlation between eosinophilia and the outcome of cord blood transplantation in adults [8]. In the present study, eosinophilia was associated with a better outcome by univariate analysis, but this was not confirmed by multivariate analysis. Since the incidence of eosinophilia differs among patients with or without CS treatment, we also analyzed its effect on prognosis in patients stratified according to systemic CS administration, but there was no significant impact of eosinophilia on the outcome in either group. Finally, multivariate analysis showed that high-risk disease, unrelated donor, and CS therapy were adverse predictors of survival with statistical significance. However, there is a limitation in that we did not treat eosinophilia and CS administration as time-dependent covariates in multivariate analyses.

Especially after allo-HSCT, systemic CS administration is done in patients who develop various complications, such as acute GVHD or pulmonary complications. Imahashi et al. reported that eosinophilia has an independent influence on the prognosis of patients with acute GVHD receiving CS treatment, but not that of patients without CS treatment [2]. Taking our results together with these findings raises the possibility that the severity of acute GVHD and CS therapy for GVHD may strongly influence transplantation outcomes regardless of eosinophilia. Furthermore, systemic CS administration is often done for allo-HSCT patients with severe complications in addition to acute GVHD, and eosinophilia may be suppressed in those patients. Our findings about non-relapse mortality in the presence or absence of CS treatment support this interpretation.

In addition to the relationship between eosinophilia and acute GVHD, there have been several reports on the pathogenesis of eosinophilia in the setting of allo-HSCT. Akhtari et al. reported that eosinophilia is observed in patients with eosinophilic pulmonary syndrome after allo-HSCT [14], but there were no specific causes of eosinophilia in our cohort.

In conclusion, eosinophilia after allo-HSCT was not related to the outcome of transplantation or the incidence of acute GVHD in patients with or without systemic CS therapy, although this study had some limitations because it was a retrospective investigation conducted at a single institution.

## Ethics

Ethics Committee Approval: Retrospective study; Informed Consent: It was taken.

## Figures and Tables

**Table 1:**
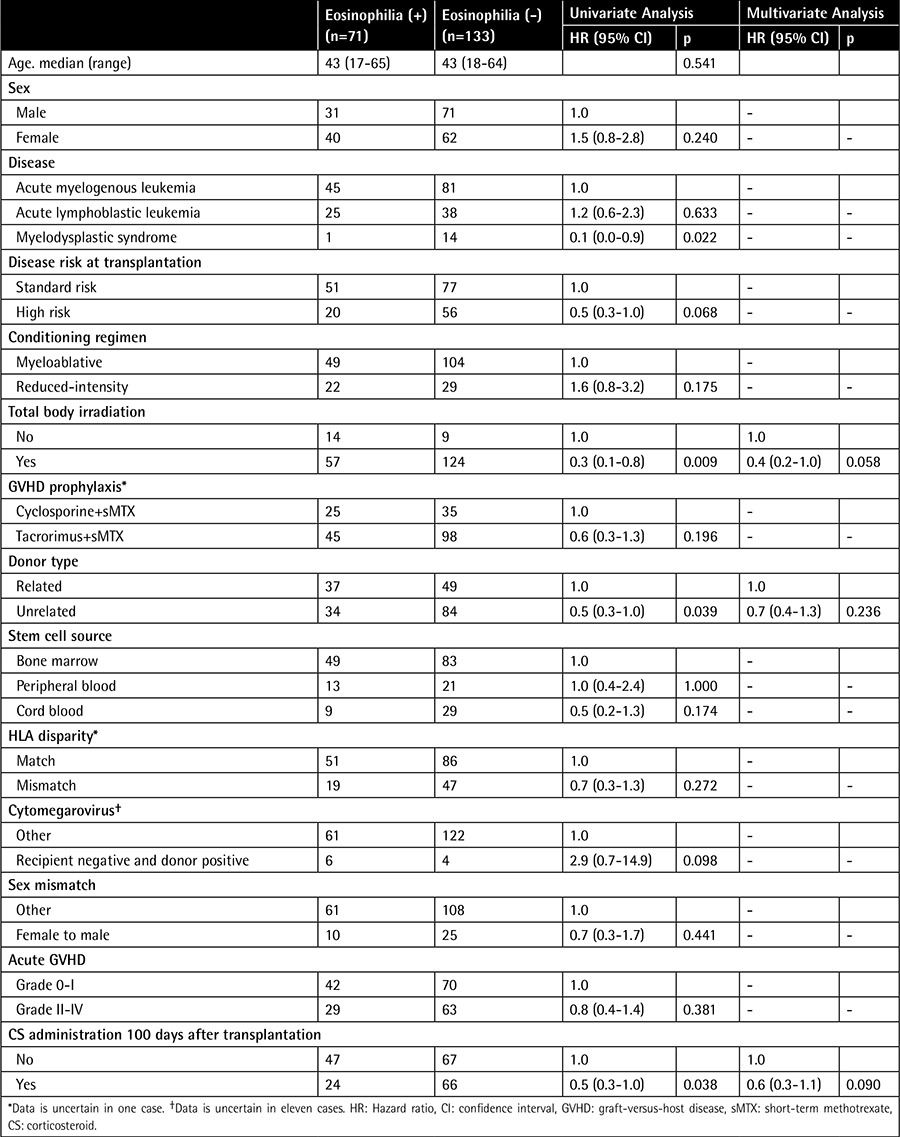
Patient characteristics and predictors of eosinophilia within 100 days after transplantation.

**Table 2:**
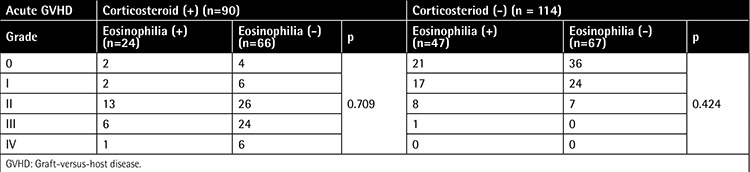
Distribution of patients with acute graft-versus-host disease.

**Table 3:**
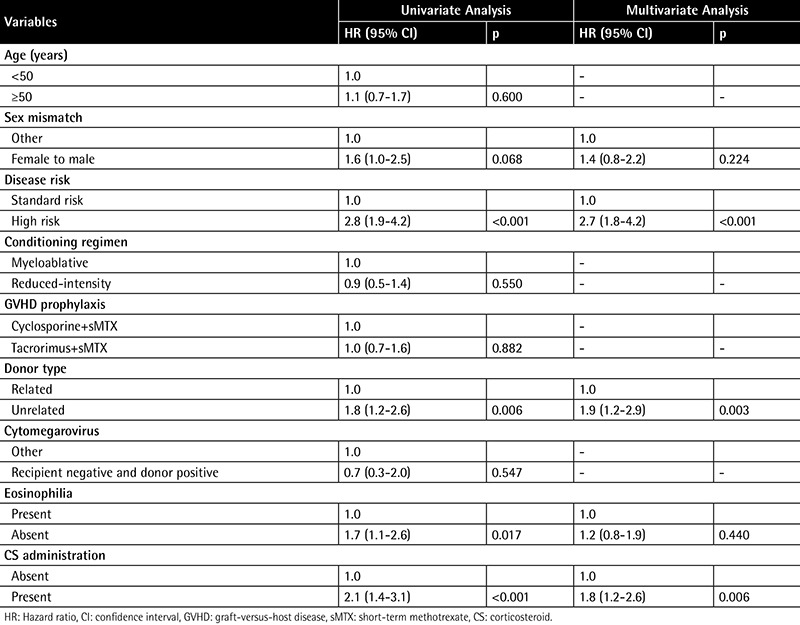
Prognostic factors for overall survival.
